# Pericarditis Caused by Hyperinvasive Strain of *Neisseria meningitidis*, Sardinia, Italy, 2015

**DOI:** 10.3201/eid2206.160160

**Published:** 2016-06

**Authors:** Cecilia Fazio, Paolo Castiglia, Andrea Piana, Arianna Neri, Maria S. Mura, Giorgia Caruana, Paola Vacca, Anna Anselmo, Andrea Ciammaruconi, Antonella Fortunato, Anna M. Palozzi, Silvia Fillo, Florigio Lista, Paola Stefanelli

**Affiliations:** Istituto Superiore di Sanità, Rome, Italy (C. Fazio, A. Neri, P. Vacca, P. Stefanelli);; Università di Sassari, Sassari, Italy (P. Castiglia, A. Piana, M.S. Mura, G. Caruana);; Centro Studi e Ricerche di Sanità e Veterinaria dell’Esercito, Rome (A. Anselmo, A. Ciammaruconi, A. Fortunato, A.M. Palozzi, S. Fillo, F. Lista)

**Keywords:** Neisseria meningitidis, bacteria, pericarditis, serogroup C, C:P1.5-1,10-8:F3-6:ST-11(cc11), hyperinvasive strain, Sardinia, Italy

**To the Editor:** Invasive meningococcal disease is usually defined by the occurrence of meningitis or septicemia. Pericarditis might occur during the course of invasive infection. This clinical picture, defined as disseminated meningococcal disease with pericarditis (*1*) or secondary meningococcal pericarditis, was reported in 1918 (*2*). In 1939, primary or isolated meningococcal pericarditis (*1**,**3*) was described. In this form of pericarditis, pericardial or blood cultures are positive for *Neisseria meningitidis* but there is no meningeal involvement or clinical meningococcemia (*4*).

Since its description, several cases of primary meningococcal pericarditis have been reported (*5*). Although its pathogenesis remains largely undefined, it has been hypothesized that the onset of primary pericarditis occurs after a transient bacteremia or as a consequence of involvement of the lower respiratory tract (*4*). Blaser et al. reported that serogroup C meningococci are usually associated with this disease, especially in adults. However, serogroups B, W, and Y have also been identified (*4*). We report a case-patient with primary meningococcal pericarditis caused by a serogroup C strain of *N. meningitidis*.

The patient was a 32-year-old man who lived in Sardinia, Italy. He had no predisposing factors, such as immunodeficiency or other chronic disorders. Disease onset occurred on August 29, 2015. Clinical manifestations were fever (temperature 38°C), hypotension, epigastralgia, arthralgia, asthenia, chest pain, and reduced vesicular murmur. The left ventricle was widely hypokinetic, and a light ST increase was observed. A blood culture was positive for *N. meningitidis.*

The patient was given piperacillin/tazobactam (4.5 g 3×/d) and metronidazole (500 mg 3×/d) for 4 days. After 4 days, treatment with ceftriaxone (2 g 2×/d) for 4 days was started. Because of persistent fever (38.8°C), levofloxacin (500 mg 2×/d) for 23 days was also started on day 7. On day 10, ceftriaxone was replaced with piperacillin/tazobactam (4.5 g 4×/d) for 21 days. A major bilateral pleural effusion was detected on the left side. On day 11, the fever had resolved. The outcome was favorable for this patient.

Drug resistance of the strain was determined by using the MIC Test Strip Method (Liofilchem, Abruzzi, Italy). Breakpoints used were those recommended by the European Committee on Antimicrobial Susceptibility Testing version 5.0 (http://www.eucast.org/). The strain was susceptible to cefotaxime, ceftriaxone, ciprofloxacin, penicillin G, and rifampin. Serogroup was determined by using slide agglutination with commercial antisera (Remel Europe, Ltd., Dartford, UK) and confirmed by PCR (*6*).

Whole-genome sequencing was conducted to obtain molecular data and enable comparison with other meningococci of the same serogroup that were isolated in Italy. Multilocus sequence typing (MLST) and typing of *porA* and *fetA* genes and Bexsero (meningcoccal group B vaccine) antigen genes (http://www.fda.gov/BiologicsBloodVaccines/Vaccines/ApprovedProducts/ucm431374.htm) were conducted as described (http://neisseria.org/). Whole-genome sequence was analyzed by using the BIGSdb Genome Comparator Tool (http://pubmlst.org/neisseria/). Genomes of meningococci belonging to the same finetype were compared by using the core genome MLST (cgMLST) approach.

The *N. meningitidis* strain of serogroup C was susceptible to all antimicrobial drugs tested. Although serogroup C was associated with 53 (41%) of 132 invasive meningococcal disease cases in Italy in 2015 (http://www.iss.it/binary/mabi/cont/Report_MBI_20151223_v4.pdf), this serogroup has not been detected in Sardinia since 2010.

Molecular analyses showed that the strain belonged to the hypervirulent clonal complex (cc) 11, sequence type (ST) 11. The complete finetype was C:5–1,10–8:F3–6:ST-11(cc11). This finetype has been reported in the United States and several countries in Europe (*7*), including Italy, and is responsible for several disease outbreaks. In Italy, this finetype represents 61% (70/115) of all serogroup C strains collected during 2012–2015. Two outbreaks caused by this strain were reported in Italy in 2007 (*8*) and in 2012 (*9*). The *N. meningitidis* factor H binding protein and heparin binding protein alleles were 1.13 and 20, respectively. The *N. meningitidis* adhesin A variant had an insertion sequence that disrupted this gene, as described for ET-15 meningococci (*10*). On the basis of results of cgMLST, the strain was determined to be related to strains responsible for an outbreak in Italy in 2015.

In summary, we report a case of meningococcal pericarditis caused by a strain of *N. meningitidis*. This strain belongs to hyperinvasive clonal complex cc11 and was identified as C:P1.5–1,10–8:F3–6:ST-11(cc11), an emerging strain in Italy and worldwide. Timely diagnosis and complete molecular characterization of this strain, which causes a rare form of invasive disease (*4*), is needed for appropriate management of patients with this disease.
